# Structural Characterization of Carbonic Anhydrase VIII and Effects of Missense Single Nucleotide Variations to Protein Structure and Function

**DOI:** 10.3390/ijms21082764

**Published:** 2020-04-16

**Authors:** Taremekedzwa Allan Sanyanga, Özlem Tastan Bishop

**Affiliations:** Research Unit in Bioinformatics (RUBi), Department of Biochemistry and Microbiology, Rhodes University, Grahamstown 6140, South Africa; asanyanga@gmail.com

**Keywords:** carbonic anhydrase VIII, single nucleotide variation, dynamic residue network analysis, MD-TASK, dynamic cross correlation

## Abstract

Human carbonic anhydrase 8 (CA-VIII) is an acatalytic isoform of the α-CA family. Though the protein cannot hydrate CO_2_, CA-VIII is essential for calcium (Ca^2+^) homeostasis within the body, and achieves this by allosterically inhibiting the binding of inositol 1,4,5-triphosphate (IP_3_) to the IP_3_ receptor type 1 (ITPR1) protein. However, the mechanism of interaction of CA-VIII to ITPR1 is not well understood. In addition, functional defects to CA-VIII due to non-synonymous single nucleotide polymorphisms (nsSNVs) result in Ca^2+^ dysregulation and the development of the phenotypes such as cerebellar ataxia, mental retardation and disequilibrium syndrome 3 (CAMRQ3). The pathogenesis of CAMRQ3 is also not well understood. The structure and function of CA-VIII was characterised, and pathogenesis of CAMRQ3 investigated. Structural and functional characterisation of CA-VIII was conducted through SiteMap and CPORT to identify potential binding site residues. The effects of four pathogenic nsSNVs, S100A, S100P, G162R and R237Q, and two benign S100L and E109D variants on CA-VIII structure and function was then investigated using molecular dynamics (MD) simulations, dynamic cross correlation (DCC) and dynamic residue network (DRN) analysis. SiteMap and CPORT analyses identified 38 unique CA-VIII residues that could potentially bind to ITPR1. MD analysis revealed less conformational sampling within the variant proteins and highlighted potential increases to variant protein rigidity. Dynamic cross correlation (DCC) showed that wild-type (WT) protein residue motion is predominately anti-correlated, with variant proteins showing no correlation to greater residue correlation. DRN revealed variant-associated increases to the accessibility of the N-terminal binding site residues, which could have implications for associations with ITPR1, and further highlighted differences to the mechanism of benign and pathogenic variants. SNV presence is associated with a reduction to the usage of Trp37 in all variants, which has implications for CA-VIII stability. The differences to variant mechanisms can be further investigated to understand pathogenesis of CAMRQ3, enhancing precision medicine-related studies into CA-VIII.

## 1. Introduction

Carbonic anhydrase related protein VIII (CARP-VIII) is an acatalytic member of the α-carbonic anhydrase (α-CA or CAH) family. This enzyme lacks CO_2_ hydration activity due to the lack of a Zn ion (Zn^2+^) coordination residue at position 116. CA-VIII contains an Arg residue substitution at position 116 as opposed to a His. His116 (His94 in CA-II) is conserved across all catalytic α-CAs, and is the first His residue responsible for the coordination of Zn^2+^ that maintains it within the active site [1]. Structurally CA-VIII comprises of 290 amino acids and has been regarded as having similar structure to the cytosolic CA-II and CA-XIII proteins [2].

Most research into the mechanism of CA-VIII has been conducted in mouse and zebrafish experiments. The human CA-VIII shares 98% and 84% sequence identity with the mouse and zebrafish proteins respectively [2]. CA-VIII is expressed highly in the cerebellum [3,4], and is essential to the development of central nervous system [5,6] and motor coordination [7]. Behavioural studies in the *wdl* mouse (19-bp deletion in CA8-gene on exon 8), and in CA-VIII knock down zebrafish models demonstrated phenotypes similar to human ataxia [8,9] highlighting significant importance to motor function.

CA-VIII functions as an inhibitor by preventing the binding of ligand inositol 1,4,5-triphosphate (IP_3_) to the IP_3_ receptor type 1 (IP_3_R1 or ITPR1) in the Purkinje cells of the cerebellum [6,10,11]. IP_3_ and ITPR1 regulate calcium (Ca^2+^) release within cells, which facilitates motor learning and synaptic plasticity [12,13,14,15,16]. Additionally, Ca^2+^ regulation is also important to nociception and the inflammatory pain response [10,11]. CA-VIII allosterically inhibits ITPR1 by reducing the receptor’s affinity for IP_3_ without altering the maximum number of ligand binding sites. It has been predicted that it achieves this by possibly altering the conformation of ITPR1 [6]. Association studies between CA-VIII and ITPR1 have found that residues 44–290 (45–291 in mouse) form the minimum binding site in CA-VIII, and interact with protein residues 1397–1657 (1387–1647 in mouse) on ITPR1 [6]. All CA-VIII residues that interact with ITPR1 are located within the CA domain (residues 27–289) [17]. Within these regions, additional research is however required to identify the exact residues essential for the binding of CA-VIII to ITPR1.

Literature investigations as to the ITPR1 domains that CA-VIII interacts with highlights a possible research gap. Research in 2003 by Hirota et al. [6], suggested that the structure of ITPR1 consisted of three domains namely; ligand binding, modulatory and channel domain. The modulatory domain has been identified as being responsible for binding numerous other cellular proteins including calmodulin (CAM) [18,19] and CA-VIII [6]. CAM like CA-VIII also assists with Ca^2+^ homeostasis in the body, and is known to bind ITPR1 residues 1564–1585, which are contained within the experimentally confirmed binding region of CA-VIII (1387–1647). In separate studies in 2002 and 2005 by Bosanac et al. [20,21], the existence of five domains comprising of the additional suppressor and coupling domain was noted. The suppressor domain was identified to be located before the ligand binding domain, and reported to bind numerous cellular proteins including CAM [21,22]. In addition, this domain was regarded as being responsible for modulating IP_3_ affinity for ITPR1 [22]. As CA-VIII and CAM play similar roles in regulating IP_3_ affinity they could potentially bind to the same region on ITPR1 (suppressor domain). Within the scope of studied literature, the binding of CA-VIII has only been investigated with respect to modulatory domain [6] and no association studies between CA-VIII and the suppressor domain have been performed.

Research into Ca^2+^ signalling has found that non-synonymous mutations to ITPR1 have been linked with cerebellar ataxia in individuals as a result of the disturbances to ITPR1 associated Ca^2+^ signalling [16,23,24,25,26,27]. Since CA-VIII has an effect on the behaviour of ITPR1, non-synonymous single nucleotide variations (nsSNVs) to CA-VIII have also been shown to have an effect on Ca^2+^ homeostasis resulting in the development of cerebellar ataxia, mental retardation and disequilibrium syndrome 3 (CAMRQ3) (MIM No: 613227). The CA-VIII nsSNVs S100P and G162R have previously been discovered to be associated with the aforementioned phenotypes [16,28,29,30,31,32]. Their treatment however poses an obstacle as the CA-VIII mechanism of action and how it interacts with ITPR1 is not well understood increasing the difficulty of drug discovery [31,33].

In the current study we investigated the effect of six nsSNVs (S100A, S100P, S100L, E109D, G162R and R237Q) on CA-VIII structure and function. As the mechanism of CA-VIII is not well understood the study was divided into two parts. Firstly, the protein structure of CA-VIII was characterised to identify binding site, and structurally and functionally important residues. Secondly, molecular dynamics (MD) simulation, dynamic cross correlation (DCC) and dynamic residue networks (DRN) analysis were used to investigate SNV effects. Binding site investigation identified 38 residues that are potentially important for CA-VIII protein-protein associations. MD analysis highlighted that variants are linked with increases to protein rigidity and compactness, with DCC showing that variant presence was associated with no correlation to greater correlated residue motion. DRN analysis provided insights as to the different mechanisms of action that benign and pathogenic variants have on CA-VIII. This research provides a foundation for the analysis of CA-VIII and ITPR1 associations. The effect of missense mutations to protein structure enhances the understanding of potential causative mechanisms of CAMRQ3 in individuals, thereby enhancing apprehension of precision medicine related studies.

## 2. Results and Discussion

The main objective of this study was to use a combination of computational approaches including MD and DRN analysis to characterise CA-VIII, and to investigate the effects of phenotype associated SNVs on protein structure and function.

### 2.1. Data Retrieval Identifies SNVs Pathogenic to CA-VIII

The Ensembl [34] and Human Mutation Analysis (HUMA) [35] databases identified three pathogenic nsSNVs and two benign SNV (see Table 1). An additional variant G162R was identified from literature studies [32]. It was noted that although G162R has been associated with CAMRQ3 [32], ClinVar and OMIM have not reported any phenotype associations. From the data in Table 1 it is observed that multiple SNVs can occur at the same position within CA-VIII and have either the same or different rs ID. For example *rs267606695* indicates two variations for residue 100; S100A and S100P. These variations have the same rs ID and demonstrate that at position 100, Ser can either be mutated to an Ala or a Pro residue. Of the six identified SNVs, VAPOR (Variant Analysis Portal) [35] (Table 1) shows that I-Mutant [36] and MUpro [37] predicted stability reduction in all. With respect to the clinical significance of the variants, S100L and E109D are regarded as benign. The results obtained for ΔΔG by the two programs MUpro and I-Mutant differ somewhat, reflecting their previously reported accuracy limits [36,37]. I-Mutant uses supported vector machines and has been trained to predict ΔΔG values. The server offers higher accuracy when 3D structures are located within the PDB (80%), however in our case since prediction was sequence based and the variants have no crystal structures within the PDB this was less accurate (77%). Correlation of experimental vs predicted mutation stability changes of 0.71 and 0.62 (structure and sequence respectively) have also been reported for the server [36]. Thus the predictions of decreased stability presented in Table 1 should be regarded as tentative. Variants S100P, G162R and R237Q are associated with CAMRQ3 [16,28,32,38,39]. The minimum allele frequency (MAF) is also presented in Table 1 and shows that all variants occur at a frequency less than 1% of the population except for E109D.

As no variant CA-VIII crystal structures exist, WT and variant proteins were modelled using MODELLER [40]. Table 1 shows the z-DOPE (normalised discrete protein energy) scores of the variant models calculated. All calculated models have a z-DOPE score less than −1.00 indicating that the variant homology models are of high quality.

**Figure 1:**
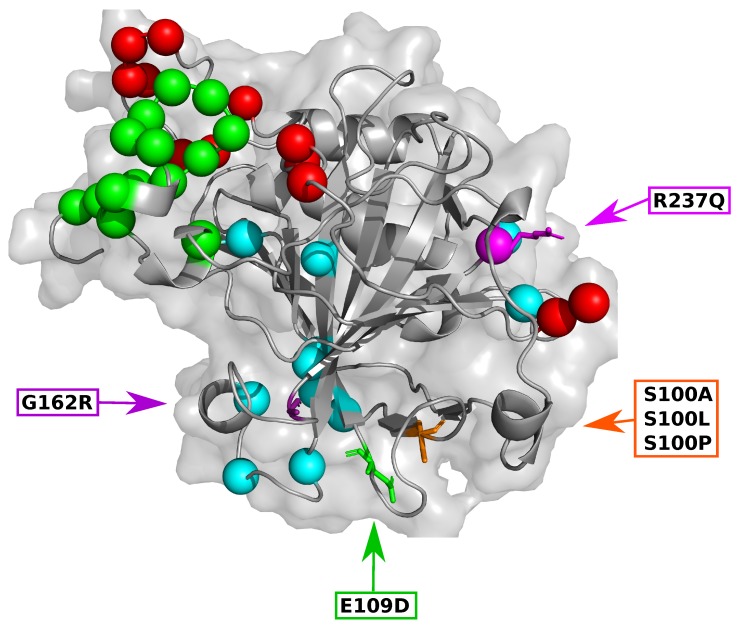
3-dimensional image of carbonic anhydrase VIII (CA-VIII) showing the SNV location, predicted binding site residues and location of each motif. **Orange**: S100A, S100L and S100P; **Green**: E109D; **Purple**: G162R; **Magenta**: R237Q. Spheres represent the binding site residues identified by SiteMap and CPORT, **Green:** Gly26, Val27, Glu28, Trp29, Gly30, Tyr31, Glu32, Glu33, Gly34, Val35, Glu36, Leu39, Val40, Ala44; **Blue:** Leu93, Lys94, Glu111, Tyr113, Arg116, Ser147, Gly151, Ile153, Asp214, Ile224, Arg237, Tyr238; **Red:** Thr255, His256, Leu262, Val263, Glu264, Gly265, Ile269, Phe274, Pro276, Gln278, Phe289, Gln290.

To further understand potential SNV effects on CA-VIII structure and function, the relationship between SNV location and protein secondary structure was investigated. Figure 1 shows the 3-dimensional (3D) SNV location on CA-VIII. The SNVs S100A, S100L and S100P are located at the end of a beta (β) sheet, while E109D, G162R and R237Q are located within a loop secondary structure. Structurally the substitution of Ser at position 100 for a Pro results in the complete destruction of the respective β-sheet and the adjacent shorter β-sheet (residues 71–73). This destruction would result in the loss of hydrogen bonds between the β-sheets, thereby having an impact on protein function and stability. Previous research [8] suggested that S100P could have an effect on the loop residues 147–162, however no variant associated structural changes were noted for these residues.

### 2.2. Functional Analysis Reveals Key Protein–Protein Interaction Residues

As previous research had identified CA-VIII residues 44–290 as the minimum binding site amino acids, SiteMap [41,42] and CPORT (Consensus Prediction Of interface Residues in Transient complexes) [43] were used to identify the potential residues participating in the association of CA-VIII with ITPR1.

SiteMap utilises an algorithm similar to Goodford’s GRID algorithm [44] whereby energetic and geometric properties are used to select site points, followed by the preparation of contour maps based on the computation of hydrophobic and hydrophilic properties at each grid point [42]. The CPORT server integrates PIER (Protein IntErface Recognition) [45], cons-PPISP (consensus Protein-Protein Interaction Site Predictor) [46], ProMate [47], SPPIDER (Solvent accessibility based Protein-Protein Interface iDEntification and Recognition) [48] and PINUP (Protein Interface residUe Prediction) [49] to predict protein-protein interaction residues.

SiteMap results revealed that only four of the top five binding sites discovered had SiteScores >0.80 (Table S1). The SiteScore represents a weighted average of the number of sites, hydrophobic and enclosure scores, and sums up-to 1.0. Binding and non-binding sites can accurately be determined by a SiteScore of 0.80. The SiteMap identified protein-protein interaction residues were located on the exterior surface of the protein across all binding sites. Each binding site discovered contained more than 30 residues, with the exception to binding site 5 that contained less than 20 residues. Binding site 1 contained the greatest number of residues. Identified binding site amino acids are presented in Table S1. Analysis of SNV positions and SiteMap data indicates that the variants are located on binding sites 2 (R237Q), 4 (S100A, S100L, E109D and S100P). Variant G162R is the only SNV not located within a binding site. The numerous binding site residues make it difficult to isolate the most important residues for protein-protein interactions, and with limited research on CA-VIII, selection of the most important binding site residues using SiteMap alone is difficult. CPORT predictions were therefore also performed to identify potential binding site residues and results are presented in Table S1. As observed with SiteMap, the binding site residues contain more than 30 amino acids. It was also noted that R237Q was the only variant located within the binding site.

To enhance residue identification, SiteMap and CPORT results were merged to obtain a consensus of the binding site residues. The residues identified by both SiteMap and CPORT were regarded as the main binding site residues for CA-VIII, and results are presented in Table S1 and Figure 1. Data shows that a consensus of 38 binding site residues were identified, with R237Q being the only SNV occurring within these residues. Results also indicate that the majority of binding site residues are located between residues 44–290, therefore agreeing with the minimum binding site residues previously discovered [6]. The data not only expands on this previous research by identifying the potential binding site residues within the range, but also identifies the residues 26–40 as also important. This observation is supported in previous literature whereby cleavage of the first 43 N-terminal residues resulted in a 16-fold decrease to CA-VIII activity [6]. Results also demonstrate that the N-terminal (green) and C-terminal (red) residues are located within close proximity to each other. In 2013 research by Aspatwar et al. [8] found that the CA-VIII region containing residues 150–157 could interact with ITPR1. Results in Table S1 and Figure 1 agree with this finding as Gly151 and Ile153 were identified as potential binding site residues. It is currently not known as to whether CA-VIII interacts with other cellular proteins, therefore the identified binding site residues could interact with proteins other than ITPR1. For this study all identified residues have been assumed to interact with ITPR1 only.

### 2.3. Sequence Analysis Identifies Residues Essential to CA-VIII Structure

Previous analysis into the acatalytic CA isoforms by Aspatwar et al. [33] demonstrated phylogenetic relationships between CA-VIII, CA-X and CA-XI. Their research however did not identify conserved residues regions important for stability and/or function within the protein. Noting this research gap, protein sequence analysis was performed to identify structurally and functionally important residue regions. Essential residues are expected to be highly conserved across different species.

Expanding on the previously identified CA-VIII binding site residues, the sequences and structures of CA-II and CA-VIII were compared. Although protein sequences share 40% identity, their 3D structural alignment shows an root mean square deviation (RMSD) difference of 1.302 Å (CA-II_*WT*_ homology model from [50], and modelled CA-VIII_*WT*_ structure) indicating structural similarity. The CA-II and CA-VIII protein sequences share 40.81% identity and their alignment is presented in Figure S1. However noting the similar structures, the sequence alignment was used to map important CA-II residues onto CA-VIII to assist in essential residue identification. Data in Table S2 presents the mapped CA-II and CA-VIII protein residues and their potential function based on the sequence alignment.

From Table S2 it is noted that the residues are divided into two groups; those that are important to catalytic function for example; CO_2_ binding site residues, active site water network residues and Zn^2+^ coordinating residues, and residues responsible for maintaining protein stability [51,52]. As CA-VIII is acatalytic the ringed amino acids were regarded as being important for the maintenance of protein stability. These residues include; Trp29, Tyr31, Trp37, Phe41, Phe117, Trp119, Phe201 and Phe250. In addition, Ser50, Leu93, Val115, Ile198 and Arg275 though not aromatic could assist with protein stability. For the remaining residues the amino acid substitutions could have other physiological functions not evident from the alignment. Previous studies have shown that the replacement of Arg116 with a His amino acid restores CO_2_ hydration activity in CA-VIII [53], and therefore these residues could be of importance then. Currently it is unknown whether the non-aromatic amino acids in Table S2 have an adaptive role in the function of acatalytic CAs, and further research is required. Interestingly, the Arg116 substitution that prevents CA-VIII from coordinating Zn^2+^ was also predicted as a potential binding site residue. This could indicate possible acatalytic adaption for Arg116. It however remains unclear as to whether the catalytic CA-VIII_*His*116_ mutant in the previous study [53] was also capable of associating with ITPR1, which would assist in the discovery of potential adaptive roles of Arg116.

### 2.4. Variant Presence Causes Conformational Changes to CA-VIII

**Figure 2:**
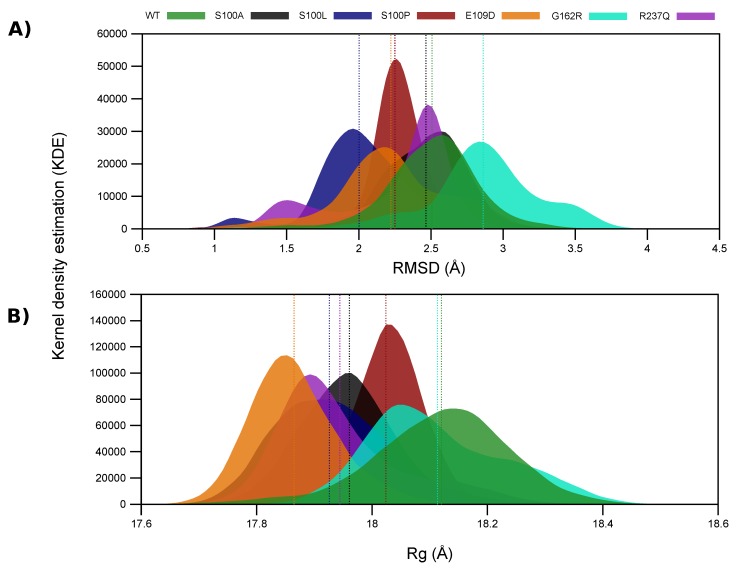
α-carbon RMSD and Rg distribution of the WT and variant proteins using the kernel density estimation. (**A**) RMSD; (**B**) Rg. The mean RMSD and Rg of the WT and variant proteins is presented as the dashed lines on each plot, and the colour of the dashed line is representative of the mean of the same coloured plot. The *x*-axis represents the sampled conformation RMSDs, and the *y*-axis peaks represent the RMSDs of the most sampled conformations.

With VAPOR results indicating stability decreases, RMSD for each MD simulation frame was calculated for the WT and variant proteins and results are presented in Figure S2A. Results demonstrate that G162R shows greater structural changes during MD simulation compared to the other proteins, suggesting potential variant instability. Results in Figure 2A present RMSD distributions demonstrating the Kernel density estimation (KDE) conformational sampling of CA-VIII during MD. The KDE is a non-parametric statistical procedure used to calculate the probability density function (PDF) of a variable. KDEs are closely related to histograms, and offer an advantage whereby there is no information loss through binning as observed in histograms. KDEs smooth the data improving interpretation allowing easier determination of distribution shape. Peaks indicate the RMSD of the most sampled protein conformation, whereas, width is indicative of the number of conformations sampled.

S100A maintains the most similar conformations to that of the WT during MD, whereas S100L exhibits the largest RMSD difference from the WT protein. As S100L is benign, this could suggest that the pathogenic effects of S100A, S100P, G162R and R237Q may not be due to global conformational changes but could be as a result of localised changes to protein residues. Though S100A, S100L and S100P all occur at the same position, Figure 2A demonstrates that S100A and S100P have the greatest structural overlap with the WT protein. It is also observed that S100L and R237Q form two distinct conformational clusters. S100L structures cluster at approximately 1.1 Å and 1.9 Å, while R237Q forms clusters at 1.5 Å and 2.5 Å. The S100L and R237Q conformations sampled at 1.1 Å and 1.5 Å are however sampled to a lesser extent. Evidence of variant associated instability of G162R is observed in Figure 2A through the presence of three potential peaks at 2.3 Å, 2.8 Å and 3.5 Å suggesting three potential major conformational clusters. The first two clusters share some structural overlap with the WT protein. The conformations sampled at 2.3 Å and 3.5 Å are however sampled at a lower frequency during MD simulation. These G162R observations are in agreement with the RMSD findings in Figure S2A.

Data in Figure 2A also shows that S100P samples less conformations than the WT and other variants (distribution width). Previous research by Turkmen et al. in 2009 [16] into the effects of S100P on CA-VIII structure and function suggested that S100P was associated with a reduction to protein stability. Further research in 2010 by Aspatwar et al. [33], suggested that substitution of a Ser by Pro at the β-sheet end (Figure 1) would result in shorter and more constrained adjacent loops as an effect of poor protein folding [33]. The poor protein folding is supported by the β-sheet destruction. More constrained loops could also explain the smaller conformational sampling and the increases to protein rigidity observed. Variant rigidity increases could have an impact on the allosteric effect of CA-VIII on ITPR1, and proteins could be too constrained to cause significant conformational changes within the receptor.

To observe the effects of SNV associated conformational sampling on protein compactness, Rg analysis was conducted and the results are presented in Figure 2B and Figure S2B. It is noted that the mutant values are smaller than that of the WT by up to 1.41% (Table 2). Although smaller Rg value may indicate increased stability, this small change does not contradict the stability prediction results presented in Table 1. Comparisons of the contributions to structural differences between the RMSD and Rg metrics are presented in Table 2 and data illustrates that the structural differences between the WT and variant CA proteins are better indicated by the RMSD.

### 2.5. WT and Variant Proteins Show Differences to Residue Movement

In the previous sections we analysed the effects of variant presence on protein conformation and compactness. To further understand SNV effects on CA-VIII structure and implications on protein conformation, residue level analysis was performed for the WT and variant proteins using dynamic cross correlation (DCC) and root mean square fluctuation (RMSF).

#### 2.5.1. DCC Analysis

As MD computes the physical motions of atoms within a protein complex during simulation [54], DCC analysis was performed to calculate the correlation between motions of the protein atoms in CA-VIII. When specific atoms move in unison they are regarded as correlated and when atoms move in opposite directions they are regarded as anti-correlated. If no relationship exists between atom movement, they are regarded as not being correlated.

**Figure 3:**
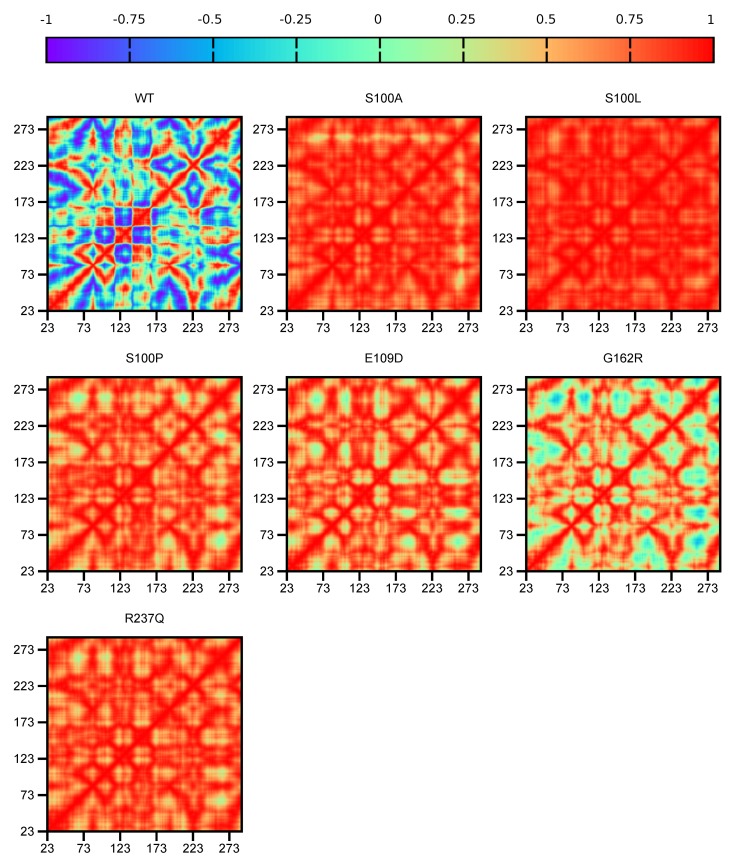
CA-VIII DCC analysis showing protein residue correlation comparison between the WT and proteins. The *x*-axis and *y*-axis represent protein residues. A value of −1 indicates that residue movement is anti-correlated, 0 highlights no movement correlation and a value of 1 represents correlated residue movement.

Data in Figure 3 presents the DCC comparison of WT and variant residues. During MD simulation the WT protein demonstrates anti-correlated movement for the majority of the residues. This anti-correlated movement could also explain the greater conformational sampling observed within the RMSD results (Figure 2). Compared to the WT protein, variant residues show greater levels of correlation to residue movement during MD simulation possibly explaining the smaller conformational sampling and increases to rigidity observed within the RMSD and Rg results.

Comparison of the variant protein data indicates that S100L residue movement is more correlated compared to the other variants. S100A, S100P, E109D, G162R and R237Q that show no correlation to motion for numerous residues, with G162R showing no correlation to the greatest extent.

The combination of DCC and the functional analysis results (see Section 2.2) assists in the identification of variant mechanism. Within the WT protein it is observed that the binding site residues move in different directions to each other (anti-correlation). The movement could be either towards each other or away from each other. This difference in movement could be necessary to facilitate the allosteric effects of CA-VIII on ITPR1. As S100A, S100L, E109D, G162R, S100P and R237Q indicate no correlation to the majority of residues, this indicates disturbances to the variant protein network. Binding to ITPR1 may produce inconsistent (random) changes to CA-VIII residue motion, and could have a detrimental effect to protein function. Compared to the other variants G162R is the only protein showing minor anti-correlation to residue movement. Though the difference between residue motion of the WT and variants is clearly evident, DCC results do not give a clear indication as to the differences to residue motion between pathogenic and benign SNVs.

Although DCC allows for the analysis of residue correlation, it does not identify relationships between residue correlation and flexibility. In the next section we analyse variant associated changes to protein flexibility.

#### 2.5.2. RMSF Analysis

**Figure 4:**
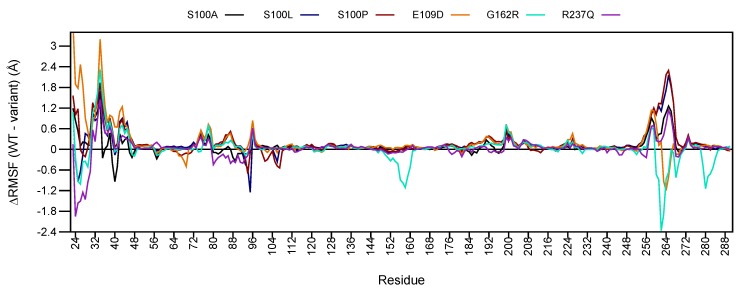
ΔRMSF (WT − variant) comparison of WT and variant proteins. Respective CA-VIII secondary structure is shown as colour coded bars at the bottom of the plot. **Red:** α-helix; **Blue:** β-sheet.

To analyse differences in residue flexibility between WT and variant proteins RMSF was determined and results are shown in Figure S3. ΔRMSF was further calculated to resolve RMSF differences between WT and variant proteins by subtracting the WT and variant RMSF data (WT − variant). These results are presented in Figure 4. A negative ΔRMSF indicates an increase to variant residue flexibility, whereas a positive ΔRMSF indicates a reduction to variant residue flexibility.

Preliminary analysis of Figure 4 shows major changes to ΔRMSF between residues 23–46 and 253–275. These regions are located within loop secondary structures therefore higher flexibility is expected, however CA-VIII binding site residues (Figure 1) are also located within these regions. At the N-terminal, binding site residues Trp29, Gly30, Tyr31, Glu32, Glu33, Gly34, Leu39 and Ala44 show reductions to residue flexibility in all variants, including Trp37. The C-terminal contains binding site residues Thr255, His256, Leu262, Val263, Glu264, Gly265, Ile269 and Phe274 which show decreases to flexibility in addition to Arg275. E109D and G162R however show increases to flexibility of these residues. In addition, G162R also shows an increase to residue flexibility between residues 150–160 which contain the predicted binding site residues Gly151 and Gly153. The increase to flexibility could have potential implications for associations with ITPR1.

Reductions to the flexibility of these residues could influence association with ITPR1. Additionally, as Trp29, Tyr31, Trp37 and Arg275 could potentially play a role in protein stability (Table S2), increases to flexibility of these residues could have an effect on CA-VIII stability, and explain the predicted stability reductions in Table 1.

Globally increases to the ΔRMSF of all variant proteins is observed for amino acids 35–48, 73–79 and 181–214. A larger number of residues show ΔRMSF values larger than zero indicating a global major reduction to protein stability. This would result in greater structural rigidity and constrainment within the protein [33], and could cause the lower conformational sampling observed within the RMSD, and the more compact structures evidenced in the Rg results. The reduction to variant residue flexibility could also explain the greater correlation observed within the variant DCC results (see Figure 3).

### 2.6. Variant Presence Is Associated with Changes to Residue Accessibility and Communication

In the previous sections RMSF and DCC highlighted at variant associated effects on the motion and flexibility of protein residues. In this section DRN analysis was used to investigate whether SNV presence has an effect on residue accessibility and communication.

Figure S4A presents the Δ*L* (change to residue accessibility) of WT and variant proteins (WT − variant). A negative Δ*L* in suggests that the variant protein residues are moving away each another and are less accessible, whereas a positive Δ*L* indicates that the residues in variant proteins are moving closer to each other and are more accessible. A Δ*L* value of 0 indicates no changes to residue accessibility between WT and variant proteins.

Comparing the variant proteins to each other, data shows that most protein residues maintain a Δ*L* close to 0. This result indicates that for the majority of the protein there are subtle to no residue accessibility changes. To identify residues showing the most significant changes to accessibility, amino acids with a Δ*L* greater than or less than two standard deviations were calculated, and results are presented in Table 3. From the data in Table 3 it is observable that majority of the amino acids comprise of the Glu rich N-terminal residues (residues 21–36) for all variants.

The variant S100L shows an unexpected result in whereby N-terminal residues show both an increase (residues 26–29 and 35) and decrease (residues 32–34) to accessibility. In addition to residues 32–34 becoming less accessible it is noted that residues 263–265 also show a reduction to accessibility. E109D Δ*L* results show a similar trend to that observed for S100L. Accessibility increases are present in binding site residues 26–29, and decreases observed in residues 31–33. With increases and decreases to accessibility occurring to residues in close proximity this could indicate the existence of a possible compensatory mechanism, whereby as one group of residues move closer together, another group moves further apart to maintain binding site integrity. This compensatory mechanism could explain the benign clinical significance (Table 1). As the green and red regions are next to each other (see Figure 1) the changes to residue accessibility could also assist with the maintenance of binding site integrity within CA-VIII.

Comparing with the pathogenic variants, results suggest that the increase and decrease to residue accessibility to maintain binding site integrity may have to occur for multiple adjacent binding site residues. Pathogenic SNVs show accessibility increases to binding site residues (residues 26–29), however this effect is not compensated for by accessibility decreases to other multiple adjacent binding site residues (residues 31–35). The Δ*L* decreases only occur to isolated residues and do not span multiple residues. Accessibility increases to Trp29 could also assist with stability maintenance in the protein.

**Figure 5:**
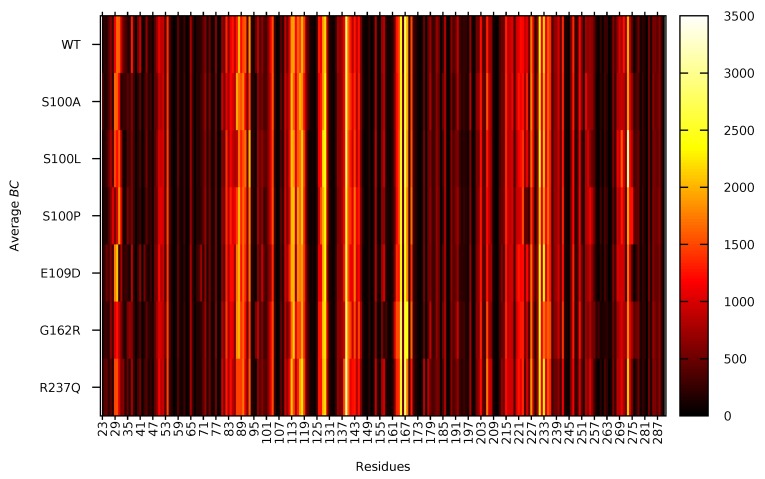
Non normalised average *BC* of the WT and variant protein residues during MD simulation.

To fully understand the changes residue accessibility could have on residue communication, average *BC* was calculated, and results are presented in Figure 5. The higher the average *BC* the more important the residue is for communication within the protein. Data in Figure 5 demonstrates that the residues; Glu139, Ile165, Ala167, Val231, Trp233 and Asn273 are the most important residues for communication within CA-VIII. Using sequence alignment (Figure S1) these amino acids map onto the CA-II residues; Glu117, Val142, Gly144, Val206, Trp208 and Asn243 which are of functional importance to CA-II (Table S2. These CA-II residues have also previously been identified as important for communication [50]. Additionally, residues Tyr113, His118, Glu128 and His129 are also associated with high average BC. Comparison of these residues with Table S2 demonstrates that His118 (His96_*CA-II*_) and Glu128 (Glu106_*CA-II*_) are of catalytic importance to CA-II. As CA-VIII is non-catalytic, this could highlight at acatalytic adaptations of these residues that could assist with the function and/or stability of CA-VIII. Tyr113 has also been identified as a potential binding site residue (Figure 1).

Data in Figure 5 shows an interesting finding whereby Asn273 in S100L is associated with the highest average *BC* of all residues in all the proteins. In addition, Trp29 and Gly30 in E109D show high average *BC* compared to the WT and other variants. Changes to the usage of these residues could indicate a compensatory measure to maintain structural stability through Trp29 and binding site integrity through Gly30.

Figure S4B presents the average Δ*BC* of the WT and variant proteins (WT − variant). An average Δ*BC* of 0 indicates that there is no change to communication of the residue within the variant protein. Positive and negative average Δ*BC* indicate a decrease and increase to the communication of variant protein residues respectively. Results in Figure S4B show that, unlike results observed with Δ*L*, numerous protein residues have Δ*BC* values greater or less than 0 suggesting that SNV presence has some effect on residue communication.

Data in Table 3 indicates the residues from Figure S4B demonstrating changes to average Δ*BC* greater than or less that two standard deviations. From the data it is observable that there are no observed accessibility and communication changes to the SNV positions apart from G162R. This suggests that substitutions at positions 100 (S100A, S100P and S100L), 109 (E109D) and 237 (R237Q) are not associated with direct changes to residue communication, indicating allosteric effects to the structure and function of CA-VIII. It is observed that the variant R237Q has the most residues showing decreases to residue communication, while G162R shows a reduction to the communication of the most binding site residues.

Analysis of the residues demonstrating a Δ*BC* increases in Table 3 highlights the possible variant mechanisms of action. Results illustrate a reduction in at least two aromatic cluster residues in all variants, with Trp37 communication reduction common in all variants. Reductions to the usage of either of the N-terminal aromatic residues Trp29, Trp37 and Phe41 could also explain the poor stability observed in previous research [16] and the development of CAMRQ3, and the stability reductions (negative ΔΔG) observed in Table 1. These amino acids are important to CA-VIII structure (Table S2. This could also explain the lack of correlation to residue movement observed in the variant DCC results (Figure 3). Lys96 though its importance to CA-VIII is yet to be determined, it is associated with a reduction to residue usage in all variants with the exception to G162R. In addition, there is also a reduction to usage of the binding site residues which could affect CA-VIII interactions with ITPR1 and result in the dysregulation of Ca^2+^ homeostasis. Interestingly, with the exception to S100P and G162R the other variants show an increase in the use of Trp29 which is responsible for stability. The increase to the residue usage could signify a compensatory measure within the other variants in order to maintain enzyme stability.

Assessment of the benign variants in Table 3 indicates that S100L and E109D are associated with usage reductions in the fewest stability maintenance residues (green and orange colours) compared to the pathogenic variants. This suggests that the benign variants do not destabilize the CA-VIII to as a great extent compared to the other variants. This is further supported by the negative ΔΔG data Table 1.

## 3. Materials and Methods

### 3.1. Software

CPORT, Bonvin Lab, Utrecht Netherlands [43]; GROMACS v2018.2, University of Groningen, Uppsala Sweden [55]; Jalview v2.10.5, The Barton Group, University of Dundee, Scotland [56]; LEaP University of California, San Francisco USA [57]; MD-TASK v1.0.1, RUBi Group Rhodes University, Grahamstown South Africa [54]; MODELLER v9.19, University of California, San Francisco USA [40]; PROPKA v3.0, Jensen Group University of Copenhagen, Copenhagen Denmark [58]; PyMOL v2.4.0, Schrödinger, New York USA [59]; SiteMap v4.8.012, Schrödinger New York, USA [41,42]; Visual Molecular Dynamics (VMD) v1.9.3, University of Illinois, Champaign USA [60].

### 3.2. Homology Modelling

#### 3.2.1. Wild-Type

The 3D structure of CA-VIII (UniProt accession P35219) has previously been solved using X-ray crystallography in the Protein Data Bank (PDB ID: 2W2J) [61]. This structure’s numbering starts from residue 23, and of the available residues some are missing atoms. Residues Glu23, Glu24, Glu28 and Glu264 are all missing the CG, CD, OE1 and OE2 atoms. Gln187 is missing the atoms CG, CD, OE1 and NE2, and Val263 is missing the CG1 and CG2 atoms.

Homology modelling using 2W2J as a template was performed to reintroduce the missing atoms to the structure. Briefly, target CA-VIII (UniProt accession number: P35219) and template (2W2J) protein sequences were aligned using MAFFT. MODELLER was then used to calculate 100 models of the CA-VIII protein and the z-DOPE score used to rank and select the models with the highest quality. The z-DOPE is a statistical method used to determine protein quality post homology modelling. The top three models with the best z-DOPE scores were then selected for further evaluation by PROSA [62] and Verify 3D [63] to validate model accuracy.

#### 3.2.2. Variants

The Ensembl genome browser was initially queried to identify CA-VIII nsSNVs. To the identified SNVs, the dbSNP database [64] was then used to select and filter only validated SNVs, followed by filtering through the Clinvar [28] database to identify SNVs containing a phenotype annotation. The HUMA (Human Mutation Analysis) VAPOR program [35] was then used to determine potential SNV effects on the stability of CA-VIII.

The identified nsSNVs were then introduced into the CA-VIII structure using homology modelling, with different structures calculated for each respective SNV. The identified SNVs were inserted into the protein by initially modifying the CA-VIII amino acid sequence to contain the selected SNVs. Homology modelling was then conducted according to the WT (Section 3.2.1) using the WT model as the template. A total of 100 models for each SNV were generated and the z-DOPE score used to select the best model.

### 3.3. Essential Residue Identification

To identify the essential CA-VIII residues, sequence alignment between the CA-VIII and CA-II (UniProt accession: P00918) proteins was performed using MAFFT [65] and results visualised in Jalview. In addition, for comparative purposes the modelled WT protein was superimposed with CA-II (CA-II_*WT*_ homology model from [50]) structure and both compared using PyMOL.

### 3.4. Identification of CA-VIII Binding Site Residues

As little literature is available as to the exact CA-VIII binding site residues essential for function, the SiteMap tool and the CPORT server were used to identify amino acids that could participate in protein–protein interactions. The modelled WT structure was prepared by protonating at pH 7.0 using PROPKA bundled with the Schrödinger Maestro Protein Preparation Wizard [66]. SiteMap site recognition was then performed to identify the potential top five binding sites by retaining sites containing a minimum of 15 site points per reported site. The search was set to use a fine grid of size 0.35 Å and restrictive hydrophobicity. From the top five sites, only binding sites with a SiteScore >0.80 were carried forward to further analysis. CPORT analysis was performed by submitting the previously prepared protein to the prediction server. WHISCY predictions were also confirmed using a very sensitive threshold and the CA-VIII FASTA file.

SiteMap and CPORT results were then collated to deduce the residues most important for CA-VIII protein–protein interaction.

### 3.5. Molecular Dynamics

Prior to molecular dynamics, all modelled CA-VIII protein structures (1 WT and 6 variants) were prepared according to Section 3.4. LEaP modelling implementing the AMBER ff14SB force field [67] was then used to generate AMBER topologies by solvating the WT and variant proteins in a 10 Å cubic box (distance between box and protein) and adding TIP3P water molecules. The solvated protein topology was then neutralised using 0.15 M NaCl. After, ACPYPE [68] was used to convert the generated AMBER topology files to GROMACS topologies.

All protein structures were then prepared for MD simulations using GROMACS. To the topologies, energy minimisation was performed using the steepest descent algorithm and until the system converged to a maximum force (F_*max*_) of 1000 kj mol^−1^ nm^−1^. Temperature (*NVT*) and pressure (*NPT*) equilibration was then performed after energy minimisation. The *NVT* ensemble was performed for 100 ps at 300 K using the Particle Mesh Ewald (PME) coulomb type for long-range electrostatics and the modified Brenson thermostat. After temperature equilibration, the *NPT* ensemble was performed at 1 bar using the Parinello–Rahman barostat algorithm [69] until the system was stable. All bonds were constrained used the LINCS algorithm during the *NVT* and *NPT* ensembles. MD simulations were performed on the Centre for High Performance Computing (CHPC) cluster using 10 CPU cores and one Nvidia Tesla v100 GPU for a time of 200 ns.

### 3.6. Molecular Dynamics Trajectory Analysis

Prior to analysis MD analysis, trajectories were stripped of water, periodic boundary conditions and centred using *cpptraj* [70]. The VMD program was then used to visualise protein dynamics and changes occurring during MD simulation. RMSD, Rg and RMSF of the protein α-carbons (C_*α*_) were then calculated using *cpptraj*.

#### Dynamic Cross Correlation (DCC)

As MD involves analysis of the physical motion of protein atoms during simulation, DCC was performed to measure the extent to which the individual CA-VIII protein atoms move together.

(1) $$C_{ij}=\frac{\langle \Delta r_{i}\cdot \Delta r_{j}\rangle }{\sqrt{\langle \Delta r_{i}^{2}\rangle }\cdot \sqrt{\langle \Delta r_{j}^{2}\rangle }}$$

DCC is calculated according to Equation (1). The Δr_i_ is the displacement of atom *i* from its average position and 〈〉 represents average time over the entire trajectory. Results are represented as a heatmap that shows the correlation of the motions C_*α*_ atoms within the protein residues over the trajectory frames.

### 3.7. Dynamic Residue Network Analysis

MD-TASK was used to perform DRN analysis of each protein. Each protein residue is a node within the DRN, and the pairwise distances between all C_*β*_ (C_*α*_ for glycine) atoms were used to determine residue interactions occurring between protein residues in each frame over the entire MD trajectory.

#### 3.7.1. Average Shortest Path (*L*)

The accessibility of a residue within a protein is defined by the average shortest path (*L*). *L* is determined by measuring total amount shortest paths to that node, and dividing by the node sum less one [54]. The formula for *L* is represented in Equation (2), where the shortest path from s to t is represented by d(s,t), the node sum is represented by n and the network nodes are represented by V.

(2) $$\alpha =\sum_{s,t\in V}\frac{d(s,t)}{n(n-1)}$$

To the MD simulation, *L* was determined using a cut of distance of 6.7 Å, using a time interval of 100 ps for every *n*th frame. *L* results were normalised on a scale of 0 to 1 to provide uniformity and then averaged to calculate average *L*. The average Δ*L* for each residue in the DRN was then calculated by subtracting the average *L* of the WT and variant proteins (WT − variant).

#### 3.7.2. *Betweenness Centrality* (*BC*)

The number of shortest paths passing through a node over the DRN defines *BC*. The greater number of paths passing through a specific node the greater its usage and the more important the residue is for protein communication. *BC* was calculated according to Equation (3), where the number of paths passing through node *v* are represented by σ(s,t|v), the network nodes are represented by *V* and the number of shortest (s,t) paths are represented as σ(s,t).

(3) $$C_{B}\left(v\right)=\sum_{s,t\in V}\frac{\sigma (s,t|v)}{\sigma (s,t))}$$

Briefly, *BC* across all MD frames was calculated and then normalised on a scale of 0 to 1 prior to averaging. Δ*BC* was then determined by subtracting WT and variant average *BC* (WT − variant).

## 4. Conclusions

In this study the function of CA-VIII was characterised and the effects of six variations (S100A, S100L, S100P, E109D and R237Q) on protein structure and function of CA-VIII investigated using MD, DCC and DRN analysis. Binding site analysis identified 38 CA-VIII residues potentially essential to the interaction of CA-VIII with other proteins and receptors. Variant analysis using MD revealed SNV-associated increases in protein rigidity and compactness. DCC analysis revealed that WT residue movement is predominately anti-correlated compared to the variants that show greater levels of residue correlation. Increases and decreases in the accessibility of adjacent N-terminal binding site residues were identified in the benign SNVs S100L and E109D, which could be a compensatory mechanism and allow the protein to tolerate the variant presence. Trp37 is associated with a usage reduction in all variants, which could have implications for CA-VIII stability. Possible future work could include Ala scanning and in-silico protein-protein docking of CA-VIII to ITPR1 to investigate the significance of binding site residues.

## Figures and Tables

**Table 1:** The rs IDs, associated residue-variant substitutions and predicted variant consequences of the identified α-carbonic anhydrase VIII single nucleotide variants (SNVs). Clinical significance data obtained from Clinvar. VAPOR was used to conduct I-Mutant and MUpro predictions.

	SNV					
	S100A	S100L	S100P	E109D	G162R	R237Q
**rs ID**	rs267606695	rs149391728	rs267606695	rs7464181	rs1421833180	rs387906598
**MAF**	0.01	<0.01	<0.01	0.50	<0.01	<0.01
**I-Mutant (ΔΔG)**	−0.66	−0.31	−0.27	−0.16	−0.84	−0.58
**MUPro (ΔΔG)**	−1.23	−0.17	−1.39	−0.41	−0.54	−1.05
**Stability**	Decrease	Decrease	Decrease	Decrease	Decrease	Decrease
**Clinical significance**	Pathogenic	Benign	Pathogenic	Benign	Pathogenic	Pathogenic
**z-DOPE score**	−1.374	−1.381	−1.41	−1.436	−1.357	−1.393

**Table 2:** Mean RMSD and Rg of the protein plots in Figure 2, and average percentage differences from the WT protein.

Protein	Metric Mean	% Difference (WT – Variant)
**RMSD**		
WT	2.507	0.00
S100A	2.465	1.68
S100L	2.002	20.14
S100P	2.251	10.21
E109D	2.222	11.37
G162R	2.862	−14.16
R237Q	2.248	10.33
**Rg**		
WT	18.120	0.00
S100A	17.961	0.88
S100L	17.926	1.07
S100P	18.024	0.53
E109D	17.865	1.41
G162R	18.113	0.04
R237Q	17.944	0.97

**Table 3:** Variant residues showing changes to Δ*L* and Δ*BC* in Figure S4. SNV positions are underlined, italicised and highlighted in bold red. Residues located within the CA-VIII binding site are underlined and highlighted in bold blue. Residues potentially important to CA-VIII stability are underlined and highlighted in bold orange. Overlapping potential protein-protein interaction and important structural residues are underlined and highlighted in bold green.

Variant Protein	Dynamic Residue Network	
	Residue Accessibility Increase (Positive Δ*L*)	Residue Communication Reduction (Positive Δ*BC*)
S100A	Glu23 Glu24 Glu25 Gly26 Val27 Glu28 Trp29 Val35 Asp43	Tyr31Glu32Trp37Val40Phe41 Thr88 Val91 Leu93 Lys96 Gly271
S100L	Glu23 Glu25 Gly26 Val27 Glu28 Trp29 Val35 Asp43	Gly30 Glu33Trp37Val40Phe41 Asp85 His87 Thr88 Lys96 Arg251
S100P	Glu23 Glu24 Glu25 Gly26 Glu28 Trp29 Gly30 Val35 Thr255	Trp29Gly30 Glu33Trp37Val40Phe41 Lys96 Gly126 Ile224 Pro225
E109D	Glu23 Glu24 Glu25 Gly26 Val27 Glu28 Trp29 Asp43 Leu70	Tyr31Trp37 Ala44 Lys96 Pro103 Val115 Ile224 Asp272
G162R	Glu23 Glu24 Glu25 Gly26 Val27 Glu28 Val35 Glu36 Leu39	Gly30Tyr31Glu32Trp37Val40Phe41 Gly86 Val91 Leu93 Val157 Ile224 Trp233 Gly271
R237Q	Glu23 Glu24 Glu25 Gly26 Val27 Trp29 Gly178	Tyr31Glu32Trp37Phe41Asp85 Thr88 Val91 Lys96 Glu114 Val115 Arg116 Ile224 Leu240 Gly271
	**Residue Accessibility Decrease (Negative** Δ ***L*** **)**	**Residue Communication Increase (Negative** Δ ***BC*** **)**
S100A	Glu32 Lys94 Lys96	Glu23 Trp29 His87 Ile89 Phe117 Leu253 Arg275
S100L	Glu32 Glu33 Gly34Trp37 Val40 Phe41 Lys96 Val263 Glu264 Gly265 Cys266 Asp267	Trp29 Glu128 Arg254 Gly268 Leu270 Asn273 Phe274 Arg275
S100P	Trp37Val40Phe41 Lys96 Pro225 Pro226	Glu28 Ser127 Ile143 Thr255 Leu270 Phe274 Arg275 Pro276
E109D	Tyr31Glu32 Glu33 Lys94 Lys96	Glu25 Trp29 Gly30 Asp43 Leu70 His87 Ile143 Gly268
G162R	Val157 Ala260 Leu262 Val263 Leu280	Val35 Glu36 His87 Arg162 Leu168 Ile234 Arg275 Pro276
R237Q	Lys94 Lys96	Trp29Phe117 Met138 Leu142 His176 Gly178 Leu206 Leu270

## Supplementary Materials

Supplementary materials can be found at https://www.mdpi.com/1422-0067/21/8/2764/s1, Table S1: CA-VIII potential protein-protein binding sites and residues SNV positions are italicised, underlined and highlighted in bold red. Figure S1: Sequence alignment showing the conserved residues between the human CA-II and CA-VIII proteins. CAH2 sequence in bold represents the reference sequence. Table S2: Mapping of CA-II residues onto the CA-VIII protein structure and predicted residue function based on the sequence alignment in Figure S1. Figure S2: α-carbon RMSD and Rg comparison between WT and variant proteins during MD simulation (**A**) RMSD; (**B**) Rg. Figure S3: RMSF comparison between the WT and variant protein residues. Figure S4: Δ*L* and Δ*BC* (WT − variant) showing changes variant residue accessibility and communication (**A**) Δ*L*; (**B**) Δ*BC*.

## Abbreviations

*BC*	*Betweenness centrality*
CA	Carbonic anhydrase
CAH	Carbonic anhydrase
CAM	Calmodulin
CARP	Cabonic anhydrase-related protein
DCC	Dynamic cross correlation
DRN	Dynamic residue network
MD	Molecular dynamics
*L*	Average shortest path
RMSD	Root mean square deviation
RMSF	Root mean square fluctuation
Rg	Radius of gyration
SNV	Single nucleotide variation
WT	Wild type
