# Supplementary material for: Structural Characterization of Carbonic Anhydrase VIII and Effects of Missense Single Nucleotide Variations to Protein Structure and Function
Source: Int J Mol Sci. 2020 Apr 16;21(8):2764. doi: 10.3390/ijms21082764 (PMC7215520; doi:10.3390/ijms21082764)
Supplement: Supplementary file 1 [file ijms-21-02764-s001.pdf]

Supplementary Materials: Structural Characterization of Carbonic Anhydrase VIII and effects of Missense Single Nucleotide Variations to Protein Structure and Function

Taremekedzwa Allan Sanyanga [1] and Özlem Tastan Bishop [1,*]

Table S1. CA-VIII potential protein-protein binding sites and residues. SNV positions are italicised, underlined and highlighted in bold red.

Binding site	SiteScore	Binding site residues
**SiteMap**		
1	0.885	23 24 25 26 27 28 29 30 31 32 33 34 35 36 37 38 39 40 41 43 44 45 46 47 49 51 52 53 54 55 58 59 60 61 62 76 77 78 79 80 81 83 84 85 86 87 88 90 92 93 94 95 96 111 113 114 116 118 119 121 122 123 124 125 126 127 128 130 133 134 135 136 137 141 143 145 152 153 154 155 157 158 159 163 165 171 172 177 178 180 181 183 184 185 187 188 190 191 192 193 194 196 198 199 200 201 202 205 222 223 224 225 226 228 229 233 245 246 248 249 251 252 254 255 256 257 258 260 261 262 263 264 266 269 270 271 272 274 275 276 277 278 279 280 281 282 283 284 285 286
2	0.908	56 57 59 60 61 65 66 69 70 72 102 131 132 134 136 169 171 210 211 212 213 214 215 216 236 ***237*** 238 239 287 288 289 290
3	0.897	43 46 47 48 64 65 67 68 102 103 104 105 106 110 158 160 161 163 164 217 218 219 220 228 229 230 231 232 282 283 284 285 286
4	0.881	68 69 71 72 73 74 75 96 97 98 ***100*** 101 106 107 ***109*** 174 175 176 177 178 204 205 207 208 209 210 212 213 238 239 240 241
5	0.792	190 191 252 254 255 256 257 258 259 260 262 263 266 267 268 269
**CPORT**		
1	NA*	26 27 28 29 30 31 32 33 34 35 36 39 40 44 93 94 111 113 116 147 151 153 214 224 ***237*** 238 255 256 262 263 264 265 269 274 276 278 289 290
**Consensus of SiteMap and CPORT**		
1	NA*	26 27 28 29 30 31 32 33 34 35 36 39 40 44 93 94 111 113 116 147 151 153 214 224 ***237*** 238 255 256 262 263 264 265 269 274 276 278 289 290

* No site scoring metric provided.

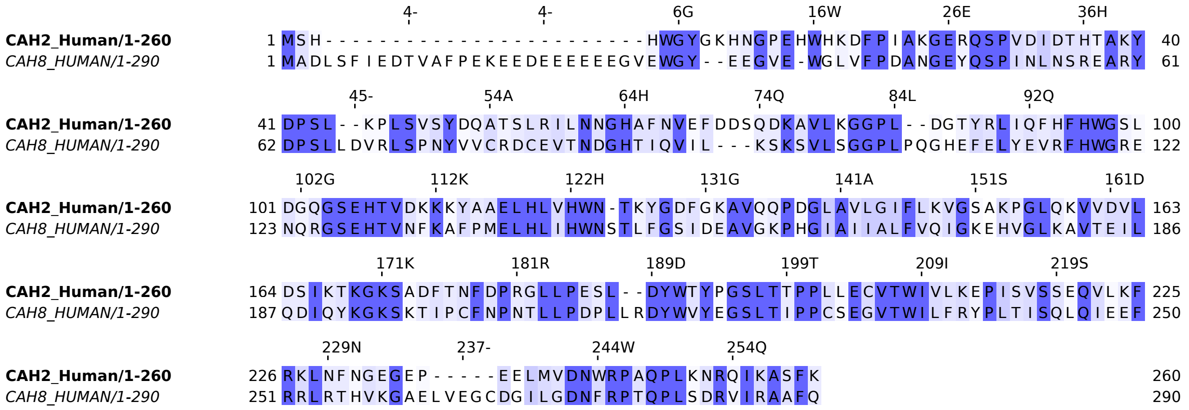

Figure S1. Sequence alignment showing the conserved residues between the human CA-II and CA-VIII proteins. CAH2 sequence in bold represents the reference sequence.

Table S2. Mapping of CA-II residues onto the CA-VIII protein structure and predicted residue function based on the sequence alignment in Figure S1.

Residue		Function	Source
**CA-II**	**CA-VIII**		
Trp5	Trp29	Aromatic cluster residue	[1–3]
Tyr7	Tyr31	Aromatic cluster residue, active site water network coordination	[1–9]
Trp16	Trp37	Aromatic cluster residue	[1–3]
Phe20	Phe41	Aromatic cluster residue	[1–3]
Ser29	Ser50	Enzymatic stability	[10,11]
Asn62	Asp85	Active site water network coordination	[4]
His64	His87	Proton shuttling residue	[1,4–8]
Phe66	Ile89	Aromatic cluster residue, secondary CO_2 binding pocket formation	[1,5–8]
Asn67	Gln90	Active site water network coordination	[12]
Phe70	Leu93	Aromatic cluster residue	[1–3]
Gln92	Glu114	Secondary Zn^{2+} ligand	[1,4]
Phe93	Val115	Aromatic cluster residue	[1–3]
His94	Arg116	Zn^{2+} coordination	[1,4]
Phe95	Phe117	Aromatic cluster residue, secondary CO_2 binding pocket formation	[1,5–8]
His96	His118	Zn^{2+} coordination	[1,4]
Trp97	Trp119	Aromatic cluster residue, secondary CO_2 binding pocket formation	[1,5–8]
Glu106	Glu128	Orientation of Zn^{2+} water ligand molecule for catalysis	[1]
Glu117	Glu139	Zn^{2+} affinity and catalytic efficiency, secondary Zn^{2+} ligand	[13]
His119	His141	Zn^{2+} coordination	[1,4]
Val121	Ile143	Primary CO_2 binding pocket formation	[1,5–8]
Val142	Ile165	Primary CO_2 binding pocket formation	[1,5–8]
Phe175	Ile198	Aromatic cluster residue	[1–3]
Phe178	Phe201	Aromatic cluster residue	[1–3]
Leu197	Leu222	Primary CO_2 binding pocket formation	[1,5–8]
Thr198	Thr223	Deep water molecule stabilisation, catalytic orientation of Zn^{2+} water ligand molecule	[4]
Thr199	Ile224	Active site water coordination, tertiary CO_2 binding pocket formation	[4]
Pro200	Pro225	Tertiary CO_2 binding pocket formation	[1,5–8]
Trp208	Trp233	Primary CO_2 binding pocket formation	[1,5–8]
Phe225	Phe250	Aromatic cluster residue, secondary CO_2 binding pocket formation	[1,5–8]
Asn243	Asn273	Tertiary CO_2 binding pocket formation, secondary Zn^{2+} ligand	[1,5–8]
Arg245	Arg275	Enzyme stability	[14]

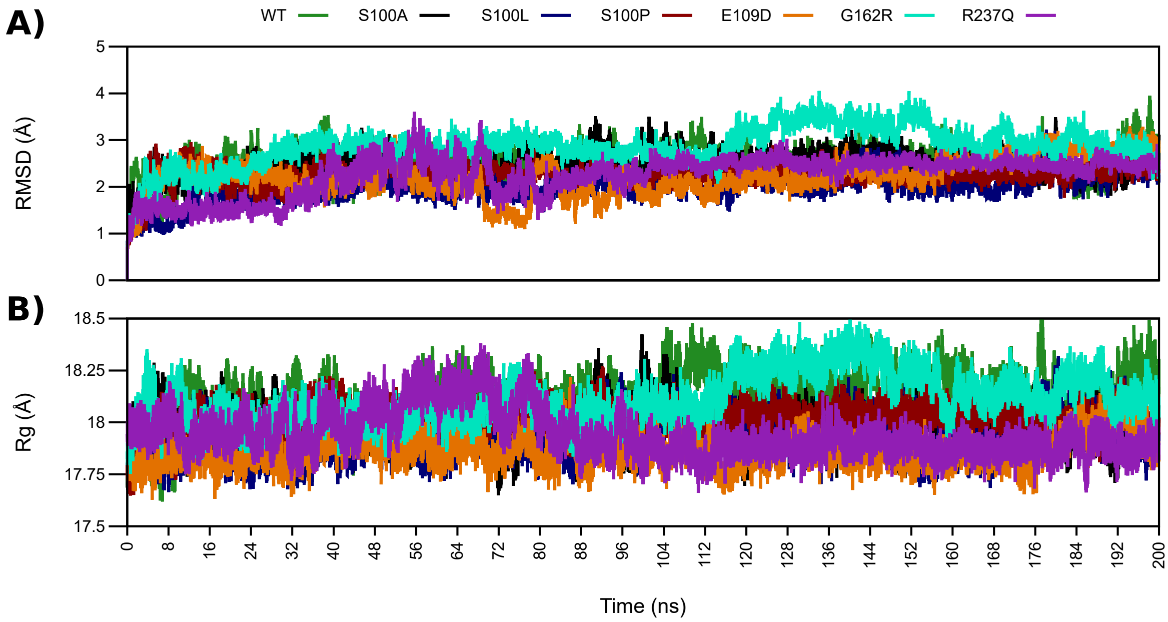

Figure S2. α-carbon RMSD and Rg comparison between WT and variant proteins during MD simulation. **(A)** RMSD; **(B)** Rg.

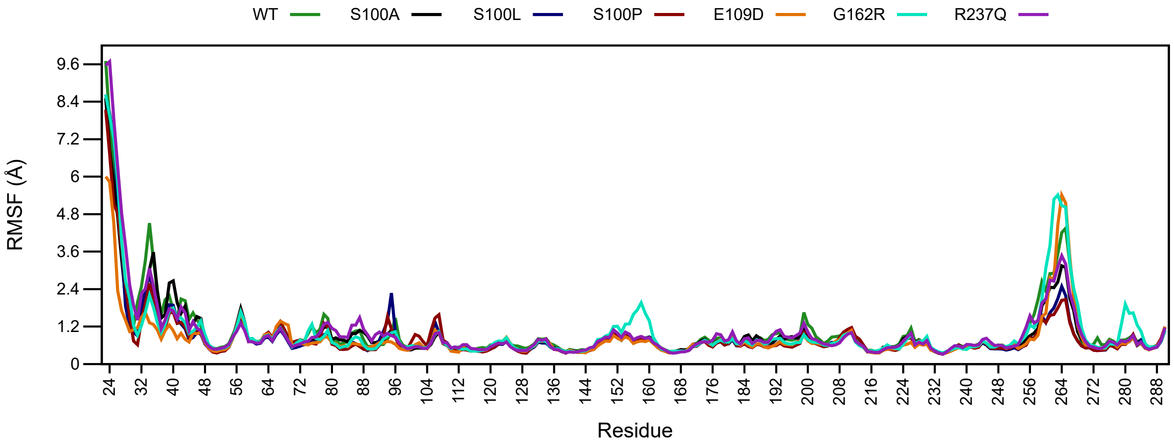

Figure S3. RMSF comparison between the WT and variant protein residues.

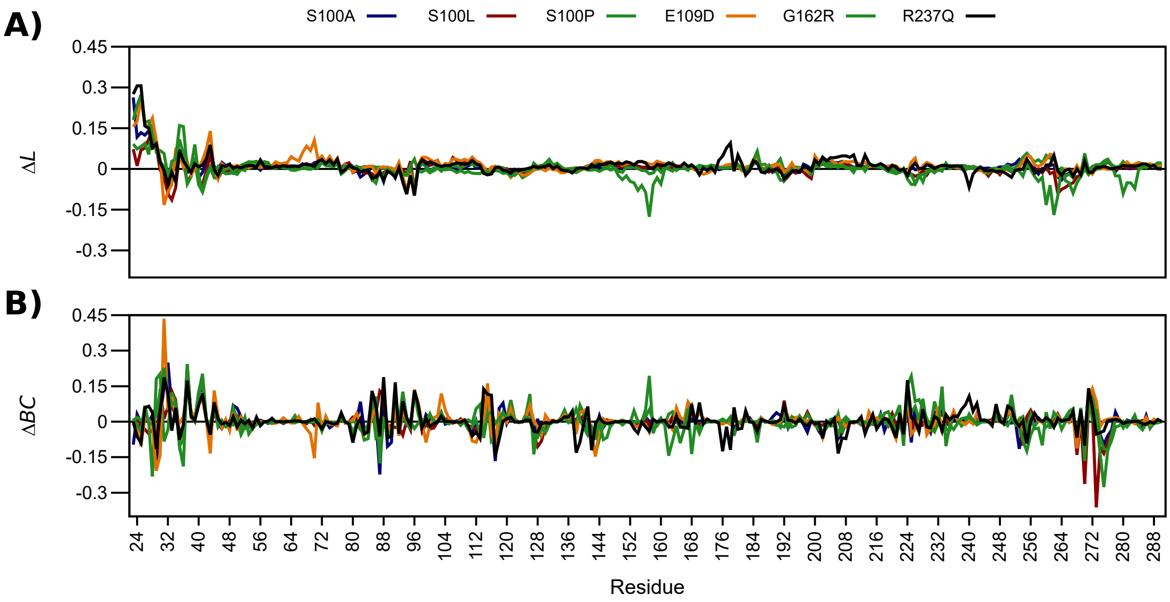

Figure S4. ΔL and ΔBC (WT − variant) showing changes variant residue accessibility and communication. **(A)** ΔL; **(B)** ΔBC.

References

1. Lindskog, S. Structure and mechanism of carbonic anhydrase. *Pharmacology & therapeutics* **1997**, *74*, 1–20.
2. Eriksson, A.E.; Jones, T.A.; Liljas, A. Refined structure of human carbonic anhydrase II at 2.0 Å resolution. *Proteins: Structure, Function, and Bioinformatics* **1988**, *4*, 274–282.
3. Hunt, J.A.; Fierke, C.A. Selection of carbonic anhydrase variants displayed on phage aromatic residues in zinc binding site enhance metal affinity and equilibration kinetics. *Journal of Biological Chemistry* **1997**, *272*, 20364–20372.
4. Silverman, D.N.; McKenna, R. Solvent-mediated proton transfer in catalysis by carbonic anhydrase. *Accounts of chemical research* **2007**, *40*, 669–675.
5. Merz Jr, K.M. Carbon dioxide binding to human carbonic anhydrase II. *Journal of the American Chemical Society* **1991**, *113*, 406–411.
6. Liang, J.Y.; Lipscomb, W.N. Binding of substrate CO2 to the active site of human carbonic anhydrase II: a molecular dynamics study. *Proceedings of the National Academy of Sciences* **1990**, *87*, 3675–3679.
7. Domsic, J.F.; Avvaru, B.S.; Kim, C.U.; Gruner, S.M.; Agbandje-McKenna, M.; Silverman, D.N.; McKenna, R. Entrapment of carbon dioxide in the active site of carbonic anhydrase II. *Journal of Biological Chemistry* **2008**, *283*, 30766–30771.
8. Alexander, R.S.; Nair, S.K.; Christianson, D.W. Engineering the hydrophobic pocket of carbonic anhydrase II. *Biochemistry* **1991**, *30*, 11064–11072.
9. Mikulski, R.L.; Silverman, D.N. Proton transfer in catalysis and the role of proton shuttles in carbonic anhydrase. *Biochimica et Biophysica Acta (BBA)-Proteins and Proteomics* **2010**, *1804*, 422–426.
10. Mårtensson, L.G.; Jonsson, B.H.; Andersson, M.; Kihlgren, A.; Bergenhem, N.; Carlsson, U. Role of an evolutionarily invariant serine for the stability of human carbonic anhydrase II. *Biochimica et Biophysica Acta (BBA)-Protein Structure and Molecular Enzymology* **1992**, *1118*, 179–186.
11. Almstedt, K.; Lundqvist, M.; Carlsson, J.; Karlsson, M.; Persson, B.; Jonsson, B.H.; Carlsson, U.; Hammarström, P. Unfolding a folding disease: folding, misfolding and aggregation of the marble brain syndrome-associated mutant H107Y of human carbonic anhydrase II. *Journal of molecular biology* **2004**, *342*, 619–633.
12. Silverman, D.N.; Lindskog, S. The catalytic mechanism of carbonic anhydrase: implications of a rate-limiting protolysis of water. *Accounts of chemical research* **1988**, *21*, 30–36.
13. Kiefer, L.L.; Paterno, S.A.; Fierke, C.A. Hydrogen bond network in the metal binding site of carbonic anhydrase enhances zinc affinity and catalytic efficiency. *Journal of the american chemical society* **1995**, *117*, 6831–6837.
14. Tashian, R.E. Genetics of the mammalian carbonic anhydrases. In *Advances in genetics*; Elsevier, 1992; Vol. 30, pp. 321–356.
